# The complete chloroplast genome of *Caltha Palustris* (Ranunculaceae)

**DOI:** 10.1080/23802359.2018.1508383

**Published:** 2018-10-03

**Authors:** Junki Lee, Yoojin Kim, Hyang Sook Chun, Kisung Kwon, Youngho Koh, Tae Sun Kang, Jung-Hwa Kang, Eun-Jeong Kim, Gyoungju Nah

**Affiliations:** a Genome Analysis Center at National Instrumentation Center for Environmental Management, Seoul National University, Seoul, Republic of Korea;; b Department of Food Science and Technology, Chung-Ang University, Ahnsung, Republic of Korea;; c New Hazardous Substances Team, National Institute of Food and Drug Safety Evaluation, Ministry of Food and Drug Safety, Ohsong, Republic of Korea;; d Hantaek Botanical Garden, Yongin, Republic of Korea

**Keywords:** *Caltha palustris*, Ranunculaceae, complete chloroplast genome, next generation sequencing

## Abstract

The complete chloroplast genome sequence of *Caltha palustris*, a species of the *Ranunculaceae* family, was characterized from the *de novo* assembly of HiSeq (Illumina Co.) paired-end sequencing data. The chloroplast genome of *C. palustris* was 155,292 bp in length, with a large single-copy (LSC) region of 84,120 bp, a small single-copy (SSC) region of 18,342 bp, and a pair of identical inverted repeat regions (IRs) of 26,415 bp. The genome contained a total of 114 genes, including 80 protein-coding genes, 30 transfer RNA (tRNA) genes, and 4 ribosomal RNA (rRNA) genes. The phylogenetic analysis of *C. palustris* with 14 related species revealed the closest taxonomical relationship with *Hydrastis canadensis* in the *Ranunculaceae* family.

The genus *Caltha* consists of approximately 16 perennial flowering plants belonging to the *Ranunculaceae* family, which habitats throughout the Northern and Southern Hemispheres (Schuettpelz and Hoot 2004). *C. palustris*, also known as marsh-marigold, has a poisonous characteristic in the leaves (Darbyshire et al. 2000). Morphologically, *C. palustris* has been indistinguishable from *Ligularia fischeri,* a widely used esculent herb in Korea. Therefore, the acquisition of chloroplast DNA information from *C. palustris* is important for future DNA barcode marker development to be distinguished from edible *L. fischeri*.

The leaves of *C. palustris* were provided from Hantaek botanical garden (www.hantaek.co.kr) in Yongin-si, Korea (37° 05′ 40.4″ N, 127° 24′ 23.7″ E) and used to construct the genomic library for Illumina paired-end (PE) sequencing. The high-quality PE reads were assembled by CLC Genomics Workbench (ver. 10.0.1, CLC QIAGEN), followed by manual curation through PE reads mapping (Kim et al. 2015). Annotation of the complete chloroplast genome was performed with GeSeq and manual corrections (Tillich et al. 2017). The complete chloroplast genome sequence of *C. palustris* was submitted to GenBank with the accession number of MG581742.

The complete chloroplast genome of *C. palustris* was 155,292 bp in length with 38.14% of G + C content, comprising a large single copy (LSC) region of 84,120 bp, a small single copy (SSC) region of 18,342 bp, and a pair of inverted repeat (IRa and IRb) regions of 26,415 bp. The genome contained 114 genes including 80 protein-coding genes, 30 tRNA genes, and 4 rRNA genes. In addition, 49 simple sequence repeats (SSR) were detected with the minimum repeat number of 10, 6, 5, 5, 5, and 5 for mono-, di-, tri-, tetra-, penta-, and hexa-nucleotides, respectively, using SSR-identification program (Beier et al. 2017).

In order to investigate the evolutionary relationship, the complete chloroplast genome sequences of *C. palustris* and 14 related species were aligned using MAFFT (ver. 7.271) (Katoh et al. 2002), followed by phylogenetic tree construction obtained from a Maximum Likelihood (ML) analysis with 1000 bootstraps using MEGA 7.0 (Kumar et al. 2016). The phylogenetic tree exhibited the close relationship of *C. palustris* with *Hydrastis canadensis* in the family of Ranunculaceae (Figure 1).

**Figure 1.**
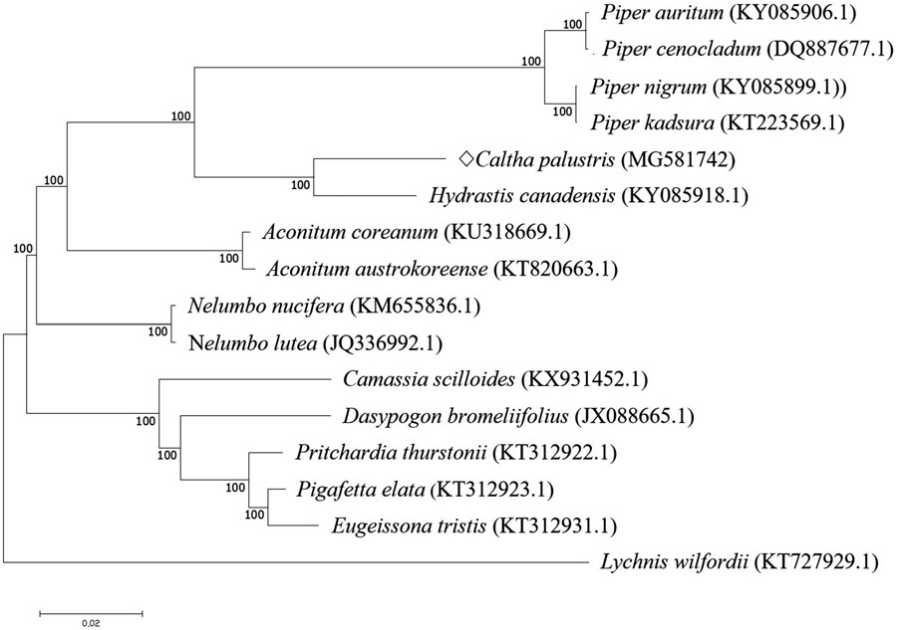
Phylogenetic analysis of *C. palustris* with 14 related species by Neighbor-Joining (NJ) methods. Phylogenetic tree was generated using complete chloroplast genome sequences, including outgroup species of *Lychnis wilfordii*. Numbers in the nodes are the bootstrap values from 1000 replicates.
